# Post-exercise Body Cooling: Skin Blood Flow, Venous Pooling, and Orthostatic Intolerance

**DOI:** 10.3389/fspor.2021.658410

**Published:** 2021-05-17

**Authors:** Afton D. Seeley, Gabrielle E. W. Giersch, Nisha Charkoudian

**Affiliations:** ^1^Thermal and Mountain Medicine Division, US Army Research Institute of Environmental Medicine, Natick, MA, United States; ^2^Oak Ridge Institute of Science and Education, Belcamp, MD, United States

**Keywords:** cold water immersion, vasoconstriction, mean arterial pressure, autonomic, heat

## Abstract

Athletes and certain occupations (e.g., military, firefighters) must navigate unique heat challenges as they perform physical tasks during prolonged heat stress, at times while wearing protective clothing that hinders heat dissipation. Such environments and activities elicit physiological adjustments that prioritize thermoregulatory skin perfusion at the expense of arterial blood pressure and may result in decreases in cerebral blood flow. High levels of skin blood flow combined with an upright body position augment venous pooling and transcapillary fluid shifts in the lower extremities. Combined with sweat-driven reductions in plasma volume, these cardiovascular alterations result in levels of cardiac output that do not meet requirements for brain blood flow, which can lead to orthostatic intolerance and occasionally syncope. Skin surface cooling countermeasures appear to be a promising means of improving orthostatic tolerance via autonomic mechanisms. Increases in transduction of sympathetic activity into vascular resistance, and an increased baroreflex set-point have been shown to be induced by surface cooling implemented after passive heating and other arterial pressure challenges. Considering the further contribution of exercise thermogenesis to orthostatic intolerance risk, our goal in this review is to provide an overview of post-exercise cooling strategies as they are capable of improving autonomic control of the circulation to optimize orthostatic tolerance. We aim to synthesize both basic and applied physiology knowledge available regarding real-world application of cooling strategies to reduce the likelihood of experiencing symptomatic orthostatic intolerance after exercise in the heat.

## Introduction

Occupations such as firefighting and the military often require work levels, clothing and/or ambient temperature exposures that are well-beyond thermoneutral “comfort” levels that most humans would consciously choose. Nonetheless, human physiological thermoregulation is remarkably capable of regulating elevations of core body temperature (T_c_) in the face of major challenges to this system. Furthermore, autonomic regulation of blood flow, sweating and other responses during increases in internal temperature are coordinated with other essential processes to maintain normal physiological function even in environmental extremes. The goal of the present paper is to discuss the regulation of body temperature and blood pressure in a specific setting: post-exercise hyperthermia and its relationship with decreases in orthostatic tolerance (OI). We will then discuss the mechanisms by which post-exercise body cooling may be an effective countermeasure to both protect against heat illness and counteract any tendency for orthostatic intolerance that may occur in the post-exercise state.

## Part I. Thermoregulation in the Heat

Human physiological thermoregulation is controlled by reflex neural mechanisms, which are complemented by local vascular mechanisms and behavioral responses to changes in internal and/or ambient temperature. In the present discussion, we focus primarily on reflex physiological mechanisms, supplemented by information from other areas as appropriate.

The primary central controller of thermoregulation in humans and other mammals is the preoptic area of the anterior hypothalamus (PO/AH). This region contains temperature sensitive neurons that respond with changes in firing rate to their own (local brain) temperature as well as to inputs they receive from peripheral thermoreceptors (Boulant, 2006). Elevation of body temperatures are sensed by warm-sensitive neurons in the PO/AH, which are activated and elicit reflexive increases in heat dissipation mechanisms. In humans, these are primarily sweating and cutaneous vasodilation. Increases in sympathetic cholinergic activity to eccrine sweat glands results in the production and release of sweat. The evaporation of sweat from the skin absorbs heat, thus lowering skin temperature and increasing the effective thermal gradient for heat transfer from the core to the periphery, and then to the environment.

The skin circulation works in concert with sweating to increase dissipation of heat from the body during increases in body temperature. Skin blood flow in humans is controlled by two branches of the sympathetic nervous system. Sympathetic noradrenergic vasoconstrictor nerves exhibit tonic activity at rest in thermoneutral environments, whereas the sympathetic active vasodilator system is only activated during increases in internal body temperature. During heat exposure, the initial thermoregulatory response in the skin is to withdraw the activity of the vasoconstrictor system. If body temperature continues to increase, the cutaneous vasodilator system is activated (Charkoudian, 2010; Johnson et al., 2011). This latter system is responsible for 80–90% of the large increases in skin blood flow that occur with severe heat stress, that can increase to as much as 60% of cardiac output (Rowell, 1983). Importantly for the present discussion, the skin circulation is very compliant, and contains venus plexuses which augment the amount of volume in the skin when blood flow increases (Rowell, 1983). This is helpful for the purposes of heat exchange and thermoregulation but can result in a decrease in venous return and insufficient cardiac filling – particularly if a person is standing still in a hot environment after exercise (i.e., muscle pump activity has stopped).

## Part II. Blood Pressure Regulation

The autonomic nervous system in humans has a central role in the regulation of arterial pressure. The sympathetic nervous system controls heart rate, cardiac contractility and peripheral vascular resistance via cardiac and vascular innervation, respectively. Post-ganglionic sympathetic nerves innervating the heart release primarily norepinephrine, which interacts with beta-adrenergic receptors at the pacemaker cells (sinoatrial (SA) and atrioventricular (AV) nodes) and across the myocardium to increase heart rate and contractility.

In terms of human cardiovascular function, the parasympathetic nervous system is primarily limited to vagal control of heart rate. The vagus nerve releases acetylcholine at the SA and AV nodes, decreasing heart rate via a decrease in the slope of the pacemaker potential in these cells. With the onset of exercise and with assumption of upright posture, the first mechanism to increase heart rate is a withdrawal of parasympathetic activity, followed by an increase in sympathetic activity.

The arterial baroreflex is the major autonomic reflex controlling blood pressure in humans. This reflex responds primarily to changes in blood pressure sensed by changes in activity of baroreceptors located in the carotid sinus and aortic arch (Raven et al., 2006; Charkoudian and Wallin, 2014). A decrease in blood pressure elicits reflex increases in sympathetic activity and decreases in parasympathetic activity with the goal of increasing blood pressure back to baseline. An increase in blood pressure elicits the opposite reflex responses in the baroreflex. This reflex is considered to “buffer” large swings in blood pressure, which might otherwise be dangerous to the health of the individual. If we consider the “blood pressure equivalent” of Ohm’s Law,

***Mean Arterial Pressure (MAP):***

= *Cardiac Output (CO)* × *Total Peripheral Resistance (TPR)*
= *[Heart Rate (HR)* × *Stroke Volume (SV)]* × *Total Peripheral Resistance (TPR)*

We note that sympathetic and parasympathetic neural mechanisms are able to control all three of the major variables that contribute to the maintenance of a normal arterial pressure.

### Measurement of Autonomic Control of Cardiovascular Function in Humans

Direct measurement of autonomic function in humans has proven challenging and thus has driven somewhat of a reliance on directly or indirectly measured cardiovascular components (CO, HR, SV, TPR) to evaluate change in mean arterial pressure. In this section, we will briefly review some of the most common autonomic measurement techniques; the interested reader is referred to several comprehensive reviews on this topic (Charkoudian and Wallin, 2014; Hart et al., 2017; Shoemaker et al., 2018; Holwerda et al., 2020).

The gold standard for measurement of sympathetic nerve activity in humans is the technique of microneurography, developed in the late 1960s by Karl-Erik Hagbarth and colleagues at the University of Uppsala (Vallbo et al., 2004). This approach involves the use of a tungsten microelectrode, which is placed across the skin at the area of interest (usually the peroneal, median or radial nerve) and is manipulated with small movements to be close enough to the nerve of interest to record the activity of that nerve. The most common recordings of human sympathetic activity are multi-unit recordings, in which several action potentials are recorded simultaneously, allowing the investigator to observe “bursts” of activity. Each burst represents a group of action potentials associated with norepinephrine release and downstream vasoconstriction (Charkoudian and Wallin, 2014; Hart et al., 2017). The most common measurements using microneurography are of sympathetic activity to the muscle vasculature (MSNA) and sympathetic activity to the skin (SSNA). One of the limitations of microneurography is that it can only measure activity of nerves that are accessible by percutaneous placement of electrodes, and cannot be used (in humans at least) for measurement of cardiac, renal or other regional activity of the sympathetic nervous system.

The most common way to get an index of the activity of the sympathetic nervous system in humans is with a simple measure of plasma norepinephrine (NE). Under many (but not all) conditions, including rest, plasma norepinephrine is strongly correlated with directly measured activity of the sympathetic nervous system (see next). However, the concentration of norepinephrine in the plasma at a given time is the net result of release (spillover), reuptake and metabolism – so that changes in any of these could result in changes in the plasma [NE], without changes in actual sympathetic noradrenergic activity. Thus, while this approach is helpful for assessing sympathetic activity, plasma NE data should be interpreted in the context of its limitations.

A method that has received increasing attention in recent decades is the approach of using frequency (spectral) analysis of cardiovascular variables (usually heart rate variability [HRV] or blood pressure) to give insight into the activity of sympathetic or parasympathetic nerves controlling those variables (Malliani and Montano, 2002). The major attraction of this approach is that it can be completely non-invasive and relatively simple to do (many systems offer automated HRV analyses of as little as 5 min of a 3-lead electrocardiogram). The basic idea behind frequency analyses is that the parasympathetic/vagal control of heart rate can change its activity very quickly. The vagus nerve transmits signals rapidly because it is large and myelinated, and the kinetics of acetylcholine at the heart are also rapid because of the presence of acetylcholinesterase at the synaptic junction (Draghici and Taylor, 2016). The sympathetic nerves, on the other hand, are small and unmyelinated and therefore transmit impulses relatively more slowly. This is the basis for the idea that “low frequency” power of frequency analyses is associated with sympathetic activity, whereas “high frequency” power is associated with the parasympathetic system (Draghici and Taylor, 2016). Various additional permutations of these calculations (e.g., low frequency/high frequency (LF/HF) ratio, alpha index, etc.) have been put forth over time (Malliani and Montano, 2002). Unfortunately, frequency analysis has many limitations and should not be used as a replacement for more direct measurement (such as those described above). For example, the high frequency component of HRV is not consistently associated with the tachycardia associated with direct pharmacological manipulation of the vagal system using atropine (Picard et al., 2009). If these analyses are used, they should be interpreted in light of their limitations and when possible as adjuncts to other approaches (Diaz and Taylor, 2006).

## Part III. Post-Exercise Regulation of Blood Pressure and Body Temperature

The mechanisms that control thermoregulation and blood pressure are markedly challenged during exercise, particularly during exercise in the heat. Exercise increases the metabolic need for oxygen delivery at the skeletal muscle (Rowell, 1974), which is achieved via complementary mechanisms. During exercise, there is a decrease in sympathetic nerve activity of the vasoconstrictor organs (Chen and Bonham, 2010), allowing greater circulation to the working skeletal muscle to meet increased metabolic demands. These central mechanisms are aided by local vasodilator mechanisms including an increase in nitric oxide synthase activity (McNamara et al., 2014). Since dynamic exercise generates heat, it contributes to elevations in body temperature and therefore stimulates cutaneous vasodilation to a degree reflective of both elevations in skin and internal temperatures (Johnson, 2010).

Post-exercise, there is also a shift to a lower baroreflex setpoint (Halliwill et al., 2000), prompting greater venous pooling around the skeletal muscle (Halliwill 2013, Chen and Bonham, 2010) which can persist for several hours. Although this may be helpful to some aspects of post-exercise recovery, this persistent vasodilation in the periphery (which can contribute to post-exercise hypotension), tends to exacerbate orthostatic intolerance. The persistent vasodilation leads to more blood pooling in the extremities, decreasing venous return. This is particularly true immediately post-exercise when muscular contractions cease to serve as a skeletal muscle pump assisting in venous return (Rowell, 1974). Both high-intensity and endurance exercise can produce this effect of blood pooling in the skeletal muscle exacerbating orthostatic intolerances (Bjurstedt et al., 1983; Halliwill, 2001; Halliwill et al., 2013; Luttrell and Halliwill, 2015; Mundel et al., 2015). While blood pressure is markedly reduced immediately post-exercise, this hypotensive response is prolonged and in some cases has been observed to last up to 12 h (Claydon et al., 2006). Prolonged post-exercise hypotension is thought to aid in exercise recovery and adaptation. Specifically, post-exercise vasodilation, caused primarily by histamine receptor activation (Halliwill et al., 2013), may help to enhance plasma volume recovery by increasing albumin in the dilated vessels (Halliwill, 2001), allow for rapid storage of glycogen, and enhance muscle capillary density in endurance trained athletes (Halliwill et al., 2013).

### Exercise Heat Stress and Contributing Mechanisms

The mechanisms governing blood pressure and body temperature regulation are further challenged when ambient heat is added to the exercise challenge (Johnson, 2010). During exercise in the heat, cardiac output, at a point determined by both exercise intensity and degree of thermal stress, cannot increase sufficiently to fuel both the exercising skeletal muscle, and the skin to allow heat dissipation, thus, there is a competition for blood flow between these two circulations (Johnson, 2010). These cumulative demands can exacerbate post-exercise orthostatic intolerance as they contribute to a greater venous pooling in cutaneous and skeletal muscle compartments resulting from reductions in vascular resistance (Schlader et al., 2016b) effectively decreasing venous return and cerebral blood flow. For example, both elevated core and skin temperatures have been observed to reduce tolerance to lower body negative pressure (LBNP) (Pearson et al., 2017). Importantly, heat stress also leads to significant reductions in body mass reflective of sweat production and evaporation meant to dissipate heat. Sweat water loss is, at least partially, drawn from blood plasma (González-Alonso et al., 2008) further exacerbating competition for a diminished blood volume, lending to an augmented risk of orthostatic intolerance both during, and post-exercise (González-Alonso et al., 2008). Overall, when combined with heat stress, body water loss has been shown to have an additive effect on orthostatic intolerance and its symptoms (Schlader et al., 2015).

### Variability in Orthostatic Tolerance

There is extensive inter-individual variability when it comes to orthostatic intolerance, which is related to factors such as age, sex, fitness status, hydration status, and certain medications. For example, while older individuals experience orthostatic intolerance and post-exercise syncope, the mechanisms governing post-exercise circulation are different (Murrell et al., 2009). Specifically, with stroke volume reduction post-exercise, younger athletes maintained total peripheral resistance, where older athletes experienced decreased TPR suggesting a decrease of sympathetic tone in both the arterial and venous vessels with age (Murrell et al., 2009). Women also appear more susceptible to orthostatic intolerance (Ganzeboom et al., 2003; Joyner et al., 2016). This might be explained by reduced cardiac filling and subsequent stroke volume in women (Fu et al., 2004), decreased mean sympathetic nerve activity and diastolic arterial pressure coherence (Yang et al., 2012), or decreased sympathetic nerve activity with respect to vasoconstriction (Joyner et al., 2016). Additionally, fitness status impacts the mechanisms associated with post-exercise hypotension and orthostatic intolerance with aerobically fit and sedentary men experiencing similar effects of hypotension post-exercise, but via distinct mechanisms (Senitko et al., 2002).

## Part IV. Post-Exercise Cold Countermeasures to Minimize Orthostatic Intolerance

### Cardiovascular Responses to Cold Exposure

Cardiovascular responses to ambient cold at rest provide a foundational glimpse into how cold exposure might assist in efforts to improve orthostatic tolerance following exercise in the heat. Both local and whole-body responses to cooling contribute to increases in arterial pressure, primarily via their effect to increase peripheral vasoconstriction (Korhonen, 2006). Heart rate contributions to a cold-induced pressor response vary, with severe local and whole body (Korhonen, 2006) cold capable of inducing tachycardia, while mild to moderate whole body exposure induces bradycardia, likely via a baroreflex response caused by vasoconstriction (Yamazaki et al., 2000). In response to moderate skin surface cooling, sensitivity of heart rate control appears to be mediated by the arterial baroreflex rather than the carotid baroreflex, suggesting a central convergence and interaction between arterial baroreceptor and skin cold receptor afferents, predominantly in the aortic baroreflex pathway.

Adjustments in stroke volume may also contribute to the cold-induced pressor response. For example, 30 min of seated cool air (14.4°C) exposure, with minimal influence on T_c_ (≤0.6°C), contributed to an intravascular fluid shift, decreasing plasma volume by 205 mL. A shift in net filtration of plasma from the blood into the interstitium is postulated to result from an increase in capillary hydrostatic pressure as a result of increased cutaneous venomotor tone (Harrison, 1985). Heightened venous return due to peripheral vasoconstriction stimulates increased atrial stretching and therefore stimulates release of plasma atrial natriuretic peptide (ANP) (Stocks et al., 2004). Circulating ANP results in enhanced sodium and water excretion and is therefore likely to be one of the mediators of cold-induced diuresis. This diuresis reduces plasma volume in response to cold stress, with cold air capable of reducing plasma volume by 7–15% (Bass and Henschel, 1956; Young et al., 1986) and cold water immersion by 15–20% (Young et al., 1986; Deuster et al., 1989). Despite reductions in plasma volume, stroke volume tends to increase in response to cold (Raven et al., 1970; Wagner and Horvath, 1985) due to a redistribution of blood from the periphery to the thoracic circulation. Blood redistribution occurs largely in response to changes in skin temperature, with maximum cutaneous vasoconstriction elicited by skin temperatures below 31°C, and is facilitated by an increase in sympathetic release of norepinephrine capable of interacting with cutaneous alpha-adrenergic receptors (Castellani and Young, 2016). The degree to which stroke volume increases appears to be linked intimately to the severity of cold, with lower ambient temperatures associated with greater increases in stroke volume (Wagner and Horvath, 1985).

Whole-body cold water immersion (CWI) has gained popularity as a post-exercise recovery technique due to its efficacy in recovering thermoregulatory variables including T_c_ and heart rate (Young et al., 1986) compared to air. CWI is a unique stimulus as it elicits physiological responses to both cold and hydrostatic pressure. Some reports have sought to differentiate between hydrostatic pressure alone (head-out thermoneutral water immersion) vs. hydrostatic pressure plus cold exposure (head-out cold water immersion). While plasma NE, systolic blood pressure, diastolic blood pressure, and TPR decreased with neutral water immersion, an increase in these variables were seen with CWI when compared to cold air. An increase in cardiac parasympathetic activity, marked by a decrease in heart rate, was elicited in both immersion conditions compared to air, with larger response induced by CWI (Mourot et al., 2008). Therefore, it appears that despite the presence of hydrostatic pressure in both neutral and CWI, a complex modulation of autonomic response ensues with cold water.

### Influences of Cold Exposure on Orthostatic Responses

Orthostatic tolerance is typically evaluated in a controlled laboratory setting using a head-up tilt test or lower body negative pressure (Yamazaki et al., 2000; Wilson et al., 2002, 2007; Durand et al., 2004; Cui et al., 2005; Johnson et al., 2017). In normothermic environments, acute moderate orthostatic stress decreases venous return and central venous pressure. Normal baroreflex responses, outlined above, result in reflex increases in heart rate and vascular sympathetic nerve activity, increasing peripheral vasoconstriction and preventing drop in arterial pressure. When cardiovascular adjustments are complicated by the concurrent presence of hyperthermia, the fall in central venous pressure and stroke volume is greater and accompanied by a blunted increase in total peripheral resistance. This TPR attenuation may be attributed to the continued prioritization of thermoregulatory convective skin perfusion thus contributing to a reduction in arterial blood pressure (Rowell, 1993; Yamazaki and Sone, 2000).

Wilson et al. (2002) examined the effects of combining whole-body heating using a water-perfused suit (46°C) combined with 10-min 60° head-up tilt to elicit orthostatic stress. The presence of concurrent heat and head-up tilt resulted in reductions in MAP as well as cerebral blood flow velocity that were attenuated, alongside an increase in total peripheral resistance, by the imposition of 15°C skin cooling, without an appreciable change in T_c_. The increase in MAP induced by skin cooling appeared to be the result of both a decrease in heart rate alongside a more influential increase in TPR. Skin surface cooling reduced mean skin temperature during normothermic tilt to ~28.3°C (~Δ 6°C) and was able to similarly reduce mean skin temperature during heated tilt to ~29.6°C (~Δ 8.5°C). Furthermore, a cool water perfused suit, applied during 5 min of active 70° head-up tilt, similarly decreased skin temperature to 28°C. Compared to post-tilt normothermia, cooling induced a tilt response marked by greater mean arterial pressure largely attributable to an exaggerated increase in total peripheral resistance (Yamazaki et al., 2000).

A series of investigations further considered 16°C skin surface cooling as a countermeasure for orthostatic intolerance induced using progressive lower body negative pressure (LBNP). Durand et al. (2004) began LBNP at −30 mmHg for 3 min and progressively reduced LBNP until the occurrence of pre-syncopal symptoms while subjects were exposed to a cold water perfused suit. Compared to normothermia, skin surface cooling enhanced a standardized cumulative stress index (mmHg/min) by 33% indicating enhanced orthostatic tolerance. At most levels of LBNP, blood pressure during cooling was greater than during normothermia and during the early stages of LBNP, cooling attenuated a reduction in cerebral blood flow velocity. Furthermore, concentrations of plasma NE increased with skin surface cooling indicating an improvement of orthostatic tolerance modulated by an increase in sympathetic activity. Skin surface cooling before and during 5-min progressive LBNP stages (−10, −15, −20, −40 mmHg) solidified the capability of cooling to augment central blood volume and consequently central venous pressure. At low enough LBNPs (−20 and −40 mmHg), the increase in central venous pressure was reflected as an elevated stroke volume believed to contribute to the enhanced MAP induced by skin surface cooling (Cui et al., 2005). Further increasing the duration of LBNP to ~15 min at −15 and −30 mmHg confirmed a 24% increase in central venous pressure accompanied by a 17% increase in pulmonary capillary wedge pressure during 16°C skin surface cooling (Wilson et al., 2007).

The results of these studies clearly established the efficacy of whole-body skin surface cooling as a countermeasure for orthostatic intolerance. However, the feasibility of implementation of water perfused suits in real-world scenarios of orthostatic stress, which are often more reactive than preventative, is low. Johnson et al. (2017) considered the “reactive” use of 0°C water face cooling during −30 mmHg LBNP stress to offset central hypovolemia. Cooling was applied to the forehead, eyes, and cheeks using a plastic bag of ice water and was maintained during 15 min of LBNP in an effort to stimulate the trigeminal nerve and consequently increase blood pressure (Schlader et al., 2016a). Face cooling effectively increased MAP via increases in cardiac output and forearm vascular resistance.

### Real-World Application of Post-exercise Cooling

Skin surface cooling can clearly increase blood pressure; however, orthostatic stress resulting from exercise, heat, and/or a combination of both introduces additional circulatory stress, potentially complicating the effectiveness of cooling efforts. The majority of the aforementioned studies use skin surface cooling, in the absence of hyperthermia, to augment total peripheral resistance, enhance central venous return and thereby increase blood pressure. The degree to which these adjustments can be made when thermoregulatory demand remains high after the cessation of exercise is often overlooked. Logic may dictate that effectiveness in offsetting post-exercise blood pressure reduction may necessitate cooler water, greater body surface area exposure to cold, or alternative cooling media. A complicating factor in this context is the so-called “sympatholytic” effect of exercise and whole-body heat stress: vascular responses to sympathetic stimulation are blunted when compared with resting conditions (Tschakovsky et al., 2002; Wilson et al., 2002). Thus, even a strong stimulator of noradrenergic vasoconstrictor nerve activity may not elicit the degree of increased peripheral vascular resistance needed to maintain or improve arterial pressure.

Early work by Franklin et al. (1993) suggests that recovery from exercise in warm conditions (31.1°C, 53% RH), albeit only post and not during exercise, contributes to elevation of T_c_ and mean skin temperature up to 60 min after exercise cessation alongside a meaningful decrease in MAP compared to baseline (76.5 ± 2.0 vs. 81.2 ± 2.4 mmHg). In contrast, when subjects are exposed to a neutral (21.4°C, 52% RH) or cool (17°C, 58% RH) post-exercise condition, both T_c_ and mean arterial pressure tend to return to baseline levels after 60 min. The likelihood of hypotension after exercise appears to be removed with cooler recovery conditions as a function of a quickened T_c_ recovery facilitated by a significant reduction in mean skin temperature. Furthermore, this study supports the notion that thermoregulatory mechanisms do play a significant role in the persistence of peripheral vasodilation post-exercise lending to the development of lowered blood pressure.

### Cutaneous and Limb Blood Flow

As mentioned previously, persistent vasodilation post-exercise combined with the loss of the skeletal muscle pump, leads to blood pooling in the extremities, decreasing venous return and consequently arterial pressure (Rowell, 1974). For example, vastus lateralis perfusion continues to elevate above exercising levels after cessation of 40 min of treadmill running in ~24°C (Ihsan et al., 2013). This post-exercise blood flow distribution may contribute to orthostatic hypotension, expected to be further exacerbated by the presence of skin thermoregulatory perfusion. Furthermore, exposing an exercised leg to 15 min of 10°C CWI reduced vastus lateralis total hemoglobin levels, suggesting that CWI may be capable of attenuating post-exercise microvascular perfusion (Ihsan et al., 2013). While a majority of the literature commenting on changes in post-exercise perfusion focus on cold water immersion, earlier reports utilized the simple application of an ice bag and yet still demonstrated attenuation of acute post-exercise perfusion elevation and edema compared to a non-cooled control limb (Yanagisawa et al., 2004). Similarly, whole-body CWI is capable of reducing post-exercise femoral vein diameter (Peiffer et al., 2009) and conductance (Mawhinney et al., 2013, 2017), arm blood flow (Vaile et al., 2011) as well as cutaneous perfusion (Mawhinney et al., 2013, 2017), although the extent of these reductions hold a non-linear relationship with CWI temperature.

Overall, elevated skeletal muscle temperature and skin perfusion following exercise in the heat contribute to a reduction in central venous pressure and a failure of TPR to increase appropriately with upright posture, leading to orthostatic intolerance. Cooling countermeasures appear to reduce both cutaneous and muscle blood flow to elicit a redistribution from the periphery to the thoracic vasculature at least when exercise is performed in thermoneutral conditions. Limited research has indicated that a reduction in large skeletal muscle microvascular perfusion following heated exercise is possible, although it appears to be smaller in magnitude than those changes seen following exercise performed in neutral ambient conditions. To improve our understanding of the influence of cooling countermeasures to prevent cardiovascular adjustments causing orthostatic intolerance, investigations examining the extent muscle and cutaneous vascular responsiveness may be blunted in response to varied cold stimuli following exercise performed in the heat are both warranted and necessary. Furthermore, it should be acknowledged that redistribution of cutaneous blood flow centrally could influence the degree of heat dissipation from the skin in a post-exercise setting. However, with a significantly widened thermal gradient elicited by skin surface cooling combined with a large preexisting degree of cutaneous vasodilation due to increased body temperatures, meaningful reductions in heat dissipation from the skin are likely minimal.

### Cerebral Blood Flow and Oxygenation

Very few studies have specifically evaluated post-exercise cerebral blood flow modulation resultant from post-exercise cooling strategies. Post-exercise cooling may offset reductions in central venous pressure that would otherwise contribute to reductions in cerebral blood flow, reducing the risk of orthostatic intolerance. Skin surface cooling using a 15°C water-perfused suit immediately before head-up tilt induced orthostatic stress was successful in preventing the fall in cerebral blood flow velocity by increasing mean arterial pressure (Wilson et al., 2002). In contrast, other literature indicates that CWI may further reduce a pre-frontal lobe NIRS-measured index of cerebral blood volume and oxygenation following heated high-intensity exercise (Minett et al., 2014). Because reduced cerebral blood flow velocity is strongly linked to orthostatic intolerance (Novak, 2016) and methodological considerations limit the interpretation of specific regional blood volume quantifications, it is likely that post-exercise cooling efforts are capable of augmenting cerebral perfusion and consequently reducing the likelihood of orthostatic intolerance. Still, further investigation of skin surface cooling vs. cold water immersion to prevent reductions in cerebral blood flow velocity specifically following heated exercise is warranted.

### Autonomic Cardiac Control: CWI and Heart Rate Variability

The ability of water immersion to increase central venous pressure via a shift of peripheral blood into the thoracic vasculature simultaneously stimulates high arterial pressure and low cardiopulmonary pressure baroreflexes (Pump et al., 2001), which can then elicit an increase in cardiac parasympathetic (vagal) tone. Since it is difficult/impossible to directly measure cardiac autonomic activity, heart rate variability (HRV) has served as a surrogate measure to evaluate post-exercise parasympathetic activity related to water immersion, and as an index of cardiovascular and hemodynamic recovery. Water temperature appears to play a key role in the effectiveness of water immersion to influence parasympathetic reactivation. Several reports implicate cold water immersion post-exercise as a greater modulator of cardiac parasympathetic reactivation compared to neutral or warm water immersions, both when exercise is performed in thermoneutral (Al Haddad et al., 2010; Stanley et al., 2012; de Oliveira Ottone et al., 2014) as well as a heated environment (Buchheit et al., 2009; Choo et al., 2018). Further reduction of water temperature beyond 14°C does not appear to elicit a greater benefit in terms of cardiovascular recovery (Choo et al., 2018). Importantly, the limitations of HRV are discussed earlier in this review and as such future use of HRV to assess post-exercise cooling responses are best used and interpreted in conjunction with more directly mechanistic measurements.

## Conclusions and Future Directions

**Figure 1:**
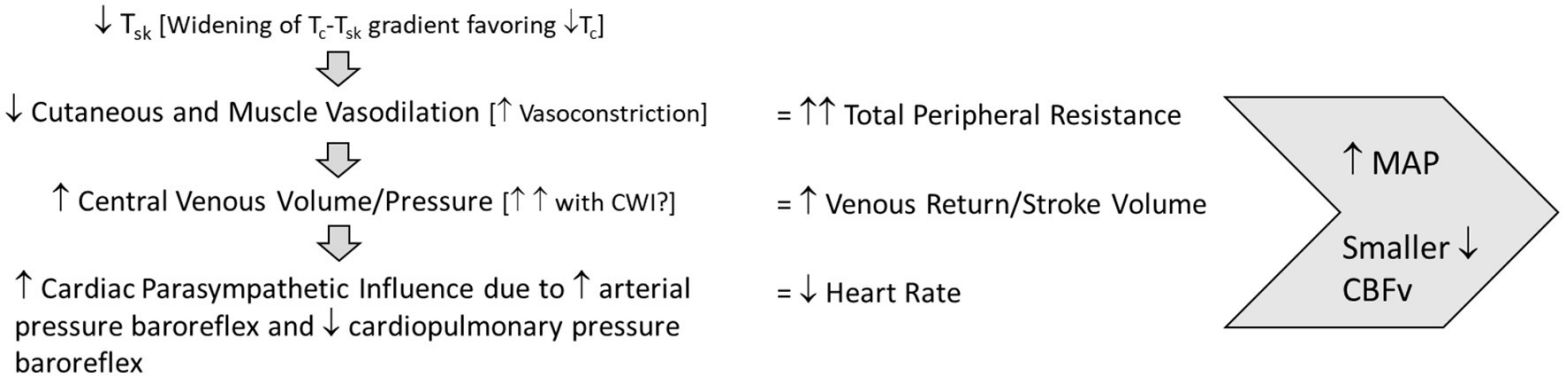
Post-exercise cooling cardiovascular adjustments to maintain orthostatic tolerance. T_*sk*_, skin temperature; T_*c*_, core temperature; MAP, mean arterial pressure; CBF_*v*_, cerebral blood flow velocity.

Successful orthostatic tolerance requires appropriate baroreflex responses to upright posture. During and after exercise in the heat, the ability of the baroreflex to cause vasoconstriction necessary to defend mean arterial pressure is limited by cutaneous vasodilation, elevated tissue temperature and peripheral venous pooling. Post-exercise cooling, especially cold water immersion, appears to augment both mean arterial pressure and cerebral vascular perfusion to minimize or prevent orthostatic intolerance after exercise in the heat (Figure 1). Still, the uniform skin temperatures created by the use of a water-perfused suit in many of the research investigations discussed within this review limit real-world applicability. Therefore, more research is necessary to further understand and optimize real-world approaches to post-exercise cooling to definitively improve orthostatic tolerance and minimize injury. Optimal timing of cooling strategies (before, during, or after exercise heat stress) to effectively offset the development of OI should also be investigated, as proactive strategies may be safer and more logistically feasible than reactive strategies. Lastly, continued evaluation of post-exercise cooling techniques specifically with women is necessary to determine the influence of estradiol and its fluctuations specifically on the cardiovascular adjustments that control skin perfusion.

## Author Contributions

All authors designed and outlined the work, performed literature reviews and interpreted findings, and drafted and revised the manuscript. All authors approved the final version of the manuscript and agree to be accountable for all aspects of the work in ensuring that questions related to the accuracy or integrity of any part of the work are appropriately investigated and resolved. All persons designated as authors qualify for authorship, and all those who qualify for authorship are listed.

## Conflict of Interest

The authors declare that the research was conducted in the absence of any commercial or financial relationships that could be construed as a potential conflict of interest.
